# A Whole Transcriptome Analysis in Peripheral Blood Suggests That Energy Metabolism and Inflammation Are Involved in Major Depressive Disorder

**DOI:** 10.3389/fpsyt.2022.907034

**Published:** 2022-05-13

**Authors:** Yu Wang, Jinxue Wei, Ting Chen, Xiao Yang, Liansheng Zhao, Min Wang, Yikai Dou, Yue Du, Rongjun Ni, Tao Li, Xiaohong Ma

**Affiliations:** ^1^Psychiatric Laboratory and Mental Health Center, West China Hospital of Sichuan University, Chengdu, China; ^2^West China Brain Research Center, West China Hospital of Sichuan University, Chengdu, China; ^3^Sichuan Clinical Medical Research Center for Mental Disorders, Chengdu, China

**Keywords:** major depressive disorder, whole transcriptome analysis, ceRNA network, KEGG pathways, co-expression modules

## Abstract

**Introduction:**

Previous studies on transcriptional profiles suggested dysregulation of multiple RNA species in major depressive disorder (MDD). However, the interaction between different types of RNA was neglected. Therefore, integration of different RNA species in transcriptome analysis would be helpful for interpreting the functional readout of the transcriptome in MDD.

**Methods:**

A whole transcriptome sequencing were performed on the peripheral blood of 15 patients with MDD and 15 matched healthy controls (HCs). The differential expression of miRNAs, lncRNAs, circRNAs, and mRNAs was examined between MDD and HCs using empirical analysis of digital gene expression data in R (edgeR). Weighted correlation network analysis (WGCNA) was used to identify RNA co-expression modules associated with MDD. A ceRNA network was constructed for interpretation of interactions between different RNA species. Kyoto Encyclopedia of Genes and Genomes (KEGG) pathway analyses were conducted to explore potential biological mechanisms associated with MDD.

**Results:**

Multiple RNAs and co-expression modules were identified to be significantly dysregulated in MDD compared to HCs. Based on the differential RNAs, a ceRNA network that were dysregulated in MDD were constructed. The pathway networks that related to oxidative phosphorylation and the chemokine signaling were found to be associated with MDD.

**Conclusion:**

Our results suggested that the processes of energy metabolism and inflammation may be involved in the pathophysiology of MDD.

## Introduction

Major depressive disorder (MDD) is a complex psychiatric disorder with high morbidity, disability rate, and great clinically heterogeneous features involving disturbances of mood and cognitive function. According to the World Health Organization (1), MDD was predicted to be the disease with the largest burden of disease in the world by 2030 (1). However, there is still a lack of biological indicators for the diagnosis and treatment of MDD. The diagnosis of MDD depends on self-reports from patients and clinical observations (2–4). Moreover, only 35–45% of patients achieved full clinical remission in the first treatment even if they received standard antidepressant treatment (5–8). Therefore, exploring the underlying mechanisms of MDD and identifying effective biomarkers are urgently required to facilitate diagnosis and treatment, which would also enhance our understanding of MDD.

Previous research reported that the prevalence of MDD demonstrated familial aggregation, which was due to the interaction between genetic effects and substantial individual-specific environmental effects, such as stressful life events, poverty and unstable intimacy (9, 10). Transcriptomics is a discipline that studies the transcription and regulation of all genes in a given cell population. The regulation of the transcriptome can effectively explain the dysregulated expression of genes in different physiological or pathological states of different individuals and is helpful for the exploration of biological markers related to physical status or diseases (11, 12). The advances in bioinformatics methods in recent years have greatly promoted the interpretation of the biological meaning of transcriptome profiles (11, 13, 14).

A number of previous studies have focused on transcriptional profiles in MDD and have shown that non-coding RNAs (ncRNAs) have potential value as biomarkers for the diagnosis and therapy of MDD (15). Accumulated evidence indicates that multiple types of RNA, including messengers (mRNAs) (16–18), microRNAs (miRNAs) (19–21), long non-coding RNAs (lncRNAs) (22, 23) and circular RNAs (circRNAs) (22, 23), are dysregulated in brain tissues, fibroblasts and peripheral blood in MDD. A correlation between RNA expression profiles and depressive symptoms was also reported (24, 25). These results suggested that alterations in transcriptome profiles might participate in the pathophysiology of MDD and be amenable for the identification of biomarkers of MDD.

The human transcriptome contains a large number of protein-coding genes as well as non-coding transcripts. Converge evidence suggests that the interaction among various types of RNA molecules plays critical roles in gene expression regulation (26). Recently, the competing endogenous RNA (ceRNA) hypothesis was proposed to interpret interactions among multiple types of RNA (27). Multiple RNA species, including mRNAs, lncRNAs and circRNAs, contain a large number of miRNA binding sites, i.e., miRNA response elements (MREs). In the ceRNA hypothesis, RNA transcripts containing MREs regulate each other by competitively binding miRNAs through shared MREs (28). Therefore, bioinformatics prediction of ceRNA networks in whole transcriptome analysis became helpful and meaningful for better interpreting the functional readout of the transcriptome (29).

In this study, a whole transcriptome sequencing was performed on the peripheral blood of patients with MDD and healthy controls (HCs) to explore the underlying molecular mechanism of MDD. The differentially expressed RNAs, dysregulation of co-expression networks, ceRNA networks and biological pathways between MDD and HCs were identified.

## Materials and Methods

### Participants

Patients were recruited from West China Hospital of Sichuan University from August 2017 to October 2018. All participants were right-handed Han Chinese and aged from 18 through 60 years. The Wechsler Adult Intelligence Scale Revised in China (WAIS-RC) was used to investigate the intelligence quotient (IQ) of participants (30, 31). To exclude the influence of medication, all patients in this study were required to be antidepressant-naive (never taken any antidepressant medication) or antidepressant-free for at least 3 months before enrollment. The depressive and anxiety symptoms of all patients were assessed using the 17-item Hamilton Depression Scale (HAMD-17) and 14-item Hamilton Rating Scale for Anxiety (HAMA-14), respectively (32, 33). The exclusion criteria for patients were (1) having serious physical diseases, such as neurogenic diseases, endocrine diseases, metabolic disorders, autoimmune diseases, cardiovascular and cerebrovascular diseases, etc.; (2) having any other psychiatric disorders in Axis I or II, such as schizophrenia, bipolar disorder, alcohol or drug abuse and history of loss of consciousness; (3) IQ ≤ 90; and (4) unsuitable or inability to complete the study for other reasons.

All HCs were recruited through advertising and were also interviewed by professional psychiatrists using SCID/NP. The exclusion criteria were as follows: (1) current and past history of depression or any psychiatric disorders in Axis I or II; (2) any psychiatric illness in their first-degree relatives; (3) serious physical diseases; (4) pregnancy or breast feeding; (5) reproductive age without adequate contraception; (6) IQ less than 90; and (4) unsuitable or unable to complete the study for other reasons.

**FIGURE 1:**
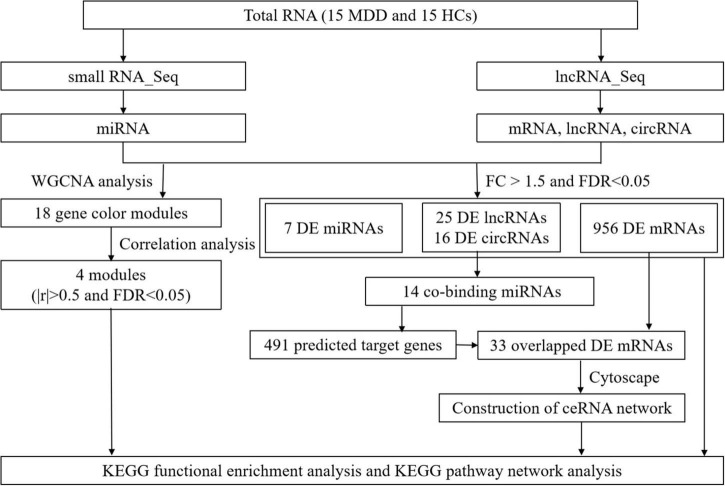
Study flow diagram. Flowchart of the comprehensive analysis process and all methods utilized in present study.

The flowchart of this study is shown in Figure 1.

### RNA Extraction and Quality Control

Peripheral blood (3 ml) samples from all participants were collected using Tempus Blood RNA Tubes (Applied Biosystems, Foster City, CA, United States), mixed vigorously for 10 s immediately and stored at –80°C until use. Total RNA was isolated using the MagMAX for Stabilized Blood Tubes RNA Isolation Kit (Thermo Fisher Scientific, Waltham, MA, United States) according to the manufacturer’s instructions. The concentration of RNA samples was determined using a NanoDrop ND-2000. The integrity of the RNA samples was assessed using a Bioanalyzer 2100.

### RNA Sequencing

One microgram of RNA was used for RNA sequencing. The mRNA, lncRNA, and circRNA in total RNA samples were enriched by removing rRNA using the Ribo-Zero™ rRNA Removal Kit (Illumina). The RNA samples were then fragmented into 200–500 nt fragments using fragmentation buffer. A reverse transcription reaction was performed to generate the first strand cDNA complementary to the fragmented RNA, and then the second strand cDNA was synthesized with incorporation of dUTP using DNA polymerase I. The double stranded cDNA was purified with a QiaQuick PCR extraction kit (Qiagen), followed by end repairing and A-tailing, and Illumina sequencing adapters were ligated to the cDNA fragments. The second strand of cDNA marked with dUTP was digested by uracil-DNA-glycosylase (UDG). The remaining strand was amplified by PCR to generate a cDNA library suitable for sequencing. RNA sequencing was performed using the NovaSeq 6000 Sequencing System (Illumina).

### Identification of mRNAs, lncRNAs, and circRNAs

Reads obtained from the sequencing machines were filtered to generate clean reads using Bowtie2 (RRID:SCR_016368). The mapped reads were assembled into transcripts using Cufflinks software (RRID:SCR_014597).

### Small RNA Sequencing

Two micrograms of RNA was used for small RNA sequencing. RNA samples were separated by polyacrylamide gel electrophoresis (PAGE), and RNA molecules in a size range of 18 ∼ 30 nt were extracted. Then, 3′ adapters and 5′ adapters were ligated to RNAs. The ligation products were reverse transcribed and amplified through PCR using primers complemented to 3′ and 5′ adapters. The amplification products with sizes of 140–160 bp were extracted to generate a cDNA library and sequenced using Illumina HiSeq™ 2500 (Illumina).

### Identification of miRNAs

Reads obtained from the sequencing machines were filtered to generate clean reads according to the following rules: (1) removing reads without 3′ adapters; (2) removing reads containing 5′ adapters; (3) removing low-quality reads containing more than one low-quality (Q value ≤ 30) base or containing unknown nucleotides; (4) removing reads with no insertion between 5′ and 3′ adapters; (5) removing reads containing poly A; and (6) removing reads shorter than 18 nt (not including adapters). The clean reads were aligned with small RNAs in the GenBank database (Release 209.0) and Rfam database (11.0) to identify and remove reads that contained sequences of rRNAs, scRNAs, snoRNAs, snRNAs, and tRNAs. Then, the reads were aligned with the reference genome to remove reads containing fragments of mRNAs. Finally, the reads were aligned with the miRBase database (Release 21) to identify miRNAs.

The lncRNA sequencing, small RNA sequencing and identification of lncRNAs, mRNAs, circRNAs and miRNAs were conducted by Gene Denove Biotechnology Co (Guangzhou, China).

### Differential Expression Analysis

The differential expression of RNAs in the comparison between MDD and HCs was conducted using the R package EdgeR (RRID:SCR_012802). First, lowly expressed RNAs with a count per million (CPM) <1 in more than 20% of the total samples were excluded. Then, a trimmed mean of M-values (TMM) normalization was performed. The biological coefficient of variation (BCV) plot was applied to examine the outliers and overall relationships of the samples. The quantile-adjusted conditional maximum likelihood (qCML) method was used for the estimation of dispersions. Differential expression was then determined using the exact test. The exact test employed in EdgeR is a method that was developed for differential expression, which is highly parallel to Fisher’s exact test. FDR < 0.05 and fold change (FC) larger than 1.5 (| log_2_FC| > 0.58) were set as thresholds for significantly differentially expressed miRNAs, mRNAs, lncRNAs, and circRNAs.

### Co-expression Module Analysis

Weighted gene co-expression network analysis (WGCNA) is a systems biology method for exploring the correlation patterns among genes across samples, which facilitates network-based identification of candidate biomarkers. A co-expression network of genes was constructed using the R package Weighted Gene co-expression Network Analysis (WGCNA, RRID:SCR_003302). The RNAs whose expression profiles were highly correlated across the samples were clustered as co-expression modules. The correlation of repression modules and MDD was then analyzed using a biweight midcorrelation method. The soft thresholding power was chosen based on the criterion of approximate scale-free topology. The minimal module size was set to 50. Highly correlated modules were merged using a cut height of 0.25. An absolute value of r larger than 0.5 was set as the threshold for a significant correlation between the expression modules and MDD.

### Construction of ceRNA Networks

The software RNAhybrid (RRID:SCR_003252) + svm_light,^1^ miRanda (RRID:SCR_017496) and TargetScan (RRID:SCR_010845) were combined for binding prediction between miRNA and lncRNA, miRNA, or mRNA. Differentially expressed lncRNAs and circRNAs were used as candidate ceRNAs. The candidate ceRNA-binding miRNAs were predicted, and the R package ceRNASeek was used to calculate ceRNA interactions (34). Then, miRNA-targeted mRNAs were predicted. Cytoscape (RRID:SCR_003032) was used for the ceRNA network visualization.

### Kyoto Encyclopedia of Genes and Genomes Pathway Analyses

The RNA transcripts identified in the differential expression analysis, co-expression module analysis and ceRNA network analysis were subjected to Kyoto Encyclopedia of Genes and Genomes (KEGG) pathway enrichment analysis. The significantly enriched pathways were subjected to KEGG pathway network analysis to identify relationships between pathways. KEGG pathway enrichment analysis and KEGG pathway network analysis were performed using the online Omicshare Tools of Gene Denove Biotechnology Co.^2^

### Statistical Analysis

Statistical analysis of demographic data was performed using IBM SPSS (version 22.0). The independent-samples *T*-test was used to compare age, education years and body mass index (BMI) between the MDD and HC groups.

## Results

### Clinical and Demographic Characteristics

Fifteen patients with MDD (6 males and 9 females) and 15 HCs (6 males and 9 females) were included in this study. There was no significant difference in age (*t* = 1.43, *p* = 0.16), education (*t* = 1.52, *p* = 0.13), or BMI (*t* = 0.68, *p* = 0.50) between MDD and HCs (Table 1). The average age of onset (year), disease duration (month), and HAMD and HAMA scores in MDD were 21.1 ± 5.2, 23.3 ± 25.3, 21.1 ± 4.2, and 14.1 ± 6.1, respectively (Table 1).

**TABLE 1:** Clinical characteristics of patients with MDD and HCs.

	MDD (*n* = 15)	HCs (*n* = 15)	*t*	*p*
Gender (male/female)	6/9	6/9	–	–
Age (year)	22.3 ± 5.2	25.0 ± 5.1	1.43	0.16
Education (years)	13.7 ± 3.3	15.4 ± 2.5	1.52	0.13
BMI	20.1 ± 2.7	20.7 ± 2.1	0.68	0.50
Age of onset (year)	21.1 ± 5.2	–	–	–
Disease duration (month)	23.3 ± 25.3	–	–	–
HAMD-17 score	21.1 ± 4.2	–	–	–
HAMA-14 score	14.1 ± 6.1	–	–	–

*MDD, major depressive disorder; HCs, health controls; HAMD, 17-item Hamilton Depression Scale; HAMA, 14-item Hamilton Anxiety Scale. Mean and standard deviation (SD) are presented in the table. Group differences were tested by analysis of Independent sample T-test, and no significant group differences were examined.*

### Summary of RNA Sequencing and Transcriptome Profiles

RNA sequencing generated 97.71 ± 7.90 G reads for MDD and 89.87 ± 23.72 G reads for HCs on average. After filtering, 96.61 ± 7.78 and 88.92 ± 23.49 G high-quality clean reads remained for MDD and HCs, respectively. There was no significant difference in raw reads and clean reads between MDD and HCs (Table 2).

**TABLE 2:** Summary of RNA sequencing data.

		MDD (*n* = 15)	HCs (*n* = 15)	*t*	*p*
Small RNA sequencing	Raw reads	12.7 ± 2.5	11.5 ± 1.7	1.53	0.13
	Clean reads	12.4 ± 2.5	11.2 ± 1.6	1.57	0.12
RNA sequencing	Raw reads	97.7 ± 7.9	89.8 ± 23.7	1.21	0.23
	Clean reads	96.6 ± 7.7	88.9 ± 23.4	1.20	0.23

*MDD, major depressive disorder; HCs, health controls; Group differences were tested by analysis of Independent sample T-test, and no significant group differences were examined.*

Small RNA sequencing generated 12.77 ± 2.57 G reads for MDD and 11.54 ± 1.75 G reads for HCs on average. After filtering, 12.49 ± 2.52 and 11.27 ± 1.68 G high-quality clean reads remained for MDD and HCs, respectively. There was no significant difference in row reads and clean reads between MDD and HCs (Table 2).

By alignment to the reference genome or miRBase database, 2,660 mature miRNAs (1,181 exist, 1,085 known and 394 novel), 26,788 lncRNAs (24,743 known and 2,045 novel), 53,545 circRNAs (11,734 exist and 41,811 novel) and 100,769 mRNA transcripts were identified, which were used for the subsequent analyses.

### Differentially Expressed RNAs in Major Depressive Disorder

**FIGURE 2:**
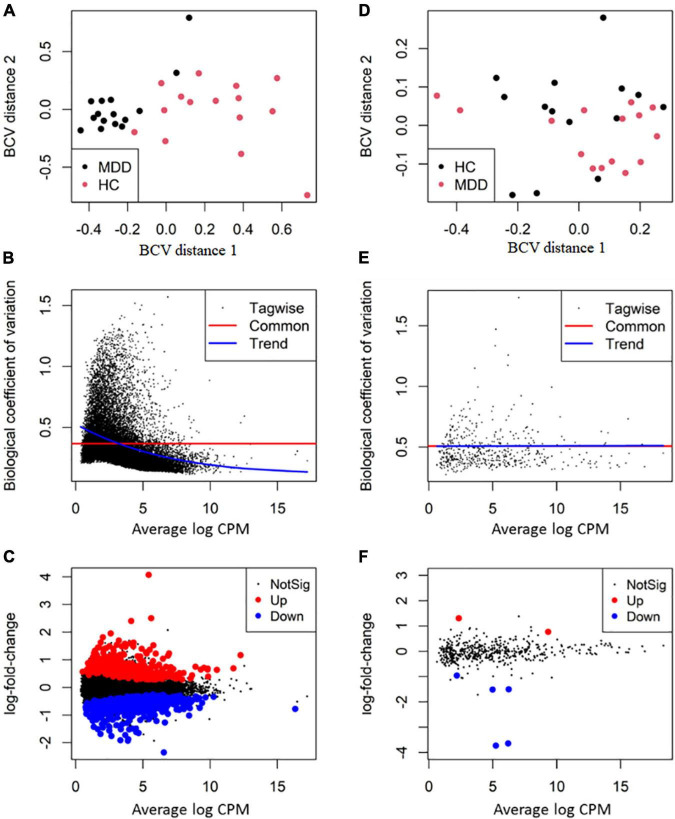
Differential expression analysis of RNA transcripts. The RNA package EdgeR was employed for differential expression analysis of RNA transcripts between major depressive disorder (MDD) and healthy controls (HCs). **(A,D)** The plot of biological coefficient of variation (BCV) distance of all the samples for RNA sequencing and small RNA sequencing, respectively; **(B,E)** the BCV plot of dispersion estimates for RNA sequencing and small RNA sequencing, respectively; **(C,F)** plots of log-fold change against log-counts per million for RNA sequencing and small RNA sequencing, respectively. The differentially expressed RNAs are highlighted.

After filtering lowly expressed RNAs, 513 miRNAs, 3,369 lncRNAs, 5,240 circRNAs and 23,453 mRNAs remained for differential expression analysis. The overall BCV plot suggested no outlier in the samples (Figures 2A,D). In the analysis of RNA sequencing data, the value of BCV was 0.39, and the common dispersion was 0.14 (Figure 2B). In the analysis of small RNA sequencing data, the value of BCV was 0.25, and the common dispersion was 0.51 (Figure 2E).

By using the exact test, 7 miRNAs (2 up-regulated and 5 down-regulated), 25 lncRNAs (20 up-regulated and 5 down-regulated), 16 circRNAs (11 up-regulated and 5 down-regulated) and 956 mRNA transcripts (439 up-regulated and 517 down-regulated) were identified as significantly dysregulated in MDD compared to HCs (Figures 2C,F). The top 10 differentially expressed miRNAs, lncRNAs, circRNAs, and mRNAs are presented in Table 3.

**TABLE 3:** Top 10 differential miRNAs, circRNAs, lncRNAs, and mRNAs between MDD and HCs.

	Transcript/ID	log2FC	*P*	FDR	Symbol
miRNA	miR-21-x	–3.643	8.83 × 10^–7^	4.65 × 10^–4^	–
	miR-1839-x	1.307	1.32 × 10^–5^	0.003	–
	hsa-miR-184	–3.729	2.22 × 10^–5^	0.003	–
	miR-125-x	–1.512	1.15 × 10^–4^	0.015	–
	hsa-miR-486-3p	–1.498	2.79 × 10^–4^	0.029	–
	miR-15-x	0.772	3.85 × 10^–4^	0.033	–
	miR-941-y	-0.959	6.43 × 10^–4^	0.048	–
	hsa-miR-1287-5p	–1.125	0.001	0.095	–
	hsa-miR-16-2-3p	–1.718	0.001	0.095	–
	miR-185-x	0.582	0.001	0.105	–
lncRNA	ENST00000508832	–2.353	2.01 × 10^–7^	2.04 × 10^–4^	MALAT1
	ENST00000619449	0.691	3.32 × 10^–6^	0.001	MALAT1
	TCONS_00044164	1.226	4.35 × 10^–6^	0.001	–
	ENST00000509654	0.747	6.62 × 10^–6^	0.001	LINC00504
	ENST00000602591	0.631	1.25 × 10^–5^	0.002	HCG18
	ENST00000510505	0.828	2.95 × 10^–5^	0.004	LINC02503
	ENST00000392385	0.604	3.20 × 10^–5^	0.004	AL590867.1
	TCONS_00096750	1.164	8.05 × 10^–5^	0.007	–
	ENST00000622604	–0.619	7.22 × 10^–4^	0.027	SMIM25
	TCONS_00096749	1.148	8.93 × 10^–4^	0.031	
circRNA	hsa_circ_001320	0.645	3.40 × 10^–5^	0.004	–
	hsa_circ_001922	0.853	1.04 × 10^–4^	0.009	–
	hsa_circ_0006382	0.707	1.48 × 10^–4^	0.011	–
	hsa_circ_000368	0.669	1.66 × 10^–4^	0.011	–
	hsa_circ_0003489	0.781	2.94 × 10^–4^	0.016	–
	novel_circ_008557	–0.671	3.29 × 10^–4^	0.017	–
	novel_circ_009744	–0.686	3.35 × 10^–4^	0.017	–
	hsa_circ_000218	0.614	5.42 × 10^–4^	0.023	–
	novel_circ_009746	–0.664	7.91 × 10^–4^	0.029	–
	hsa_circ_000373	0.724	8.88 × 10^–4^	0.031	–
mRNA	ENST00000527344	2.402	7.33 × 10^–15^	1.79 × 10^–10^	CFL1
	ENST00000596873	4.069	1.33 × 10^–10^	1.62 × 10^–6^	RPS11
	ENST00000361851	1.267	1.18 × 10^–9^	9.60 × 10^–6^	MT-ATP8
	ENST00000397043	–1.909	2.83 × 10^–9^	1.72 × 10^–5^	ATP2A3
	ENST00000539896	–1.213	4.00 × 10^–9^	1.95 × 10^–5^	PTAFR
	TCONS_00089524	1.097	4.83 × 10^–9^	1.96 × 10^–5^	Rmnd5a
	ENST00000527546	–1.516	7.93 × 10^–9^	2.76 × 10^–5^	MICAL2
	ENST00000497259	1.695	2.04 × 10^–8^	6.21 × 10^–5^	ARHGAP25
	ENST00000564304	0.903	3.29 × 10^–8^	8.35 × 10^–5^	ATXN2L
	ENST00000645079	–0.894	3.50 × 10^–8^	8.35 × 10^–5^	RPL15

### Identification of Differential Co-expression Modules

**FIGURE 3:**
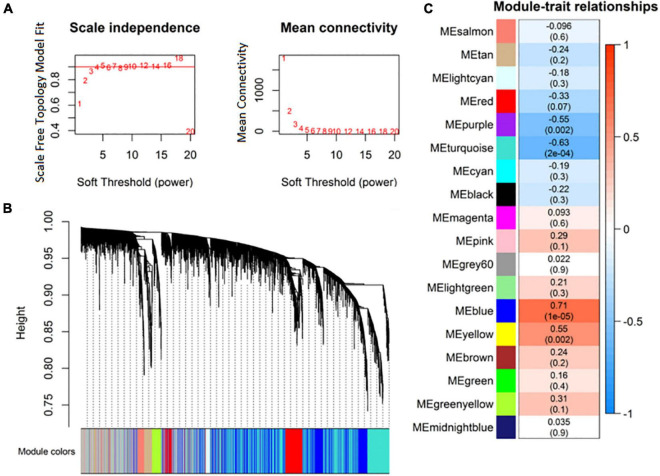
Identification of co-expression modules of RNA transcripts. **(A)** Analysis of RNA network topology for various soft-thresholding powers; **(B)** RNA expression module clustering dendrograms based on topological overlap; **(C)** association of RNA expression modules with major depressive disorder (MDD).

Highly correlated RNAs in expression profiles were clustered as expression modules using the R package WGCNA. Based on the criterion of approximate scale-free topology, 4 was selected as the soft thresholding power (Figure 3A). Eighteen expression modules of RNAs were identified (Figure 3B). Then, the correlation between the expression modules and MDD was analyzed. The purple (*r* = –0.55), turquoise (*r* = –0.63), blue (*r* = 0.71), and yellow (*r* = 0.55) modules were significantly correlated with MDD (Figure 3C).

### ceRNA Networks

**FIGURE 4:**
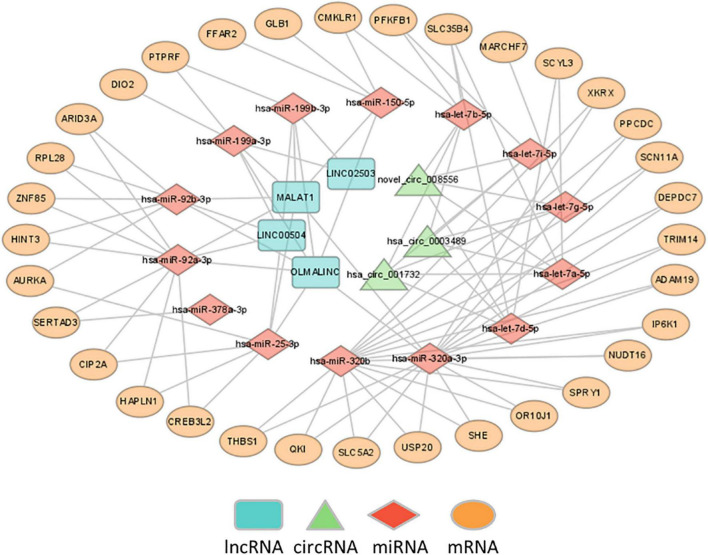
Construction of ceRNA networks. A ceRNA network was constructed using 7 ceRNAs (3 circRNAs and 4 lncRNAs), 14 miRNAs and 33 mRNA transcripts.

The differentially expressed lncRNAs and circRNAs were subjected to ceRNA interaction analysis. Four lncRNAs and three circRNAs were identified to interact and used as ceRNAs for ceRNA network construction. The ceRNA network indicated that 7 ceRNAs may bind to 14 miRNAs and regulate the expression of 33 mRNAs (Figure 4).

### Kyoto Encyclopedia of Genes and Genomes Pathway Analysis

**FIGURE 5:**
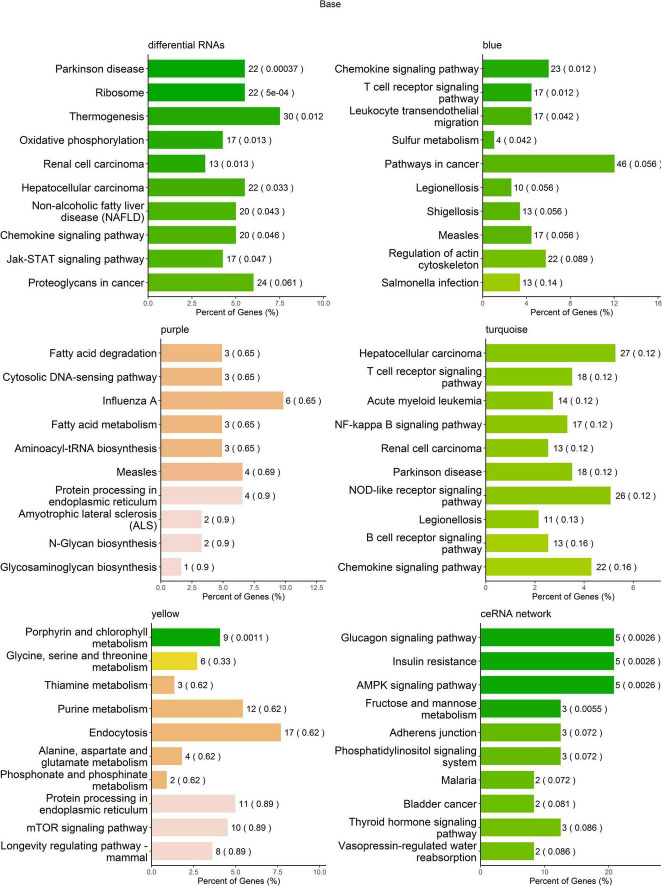
Kyoto Encyclopedia of Genes and Genomes (KEGG) pathway enrichment analysis of RNA transcripts. The top 10 pathways in KEGG pathway enrichment analysis.

To help interpret differential RNA transcript profiles in the context of biological processes between MDD and HCs, we performed KEGG pathway analysis. The results showed that the differentially expressed RNAs were enriched in multiple pathways, i.e., Parkinson’s disease, ribosome, thermogenesis, oxidative phosphorylation, renal cell carcinoma, hepatocellular carcinoma, non-alcoholic fatty liver disease (NAFLD), chemokine signaling pathway, and Jak-STAT signaling pathway (Figure 5A and Supplementary Table 1). The RNA transcripts in the blue co-expression module were enriched in four pathways, i.e., chemokine signaling pathway, T-cell receptor signaling pathway, leukocyte transendothelial migration, and sulfur metabolism; the RNA transcripts in the yellow co-expression module were enriched in the porphyrin and chlorophyll metabolism pathways. No RNAs in the turquoise and purple modules were found to be significantly enriched in KEGG pathways (Figures 5B–E and Supplementary Table 2). The mRNAs regulated by ceRNA networks were enriched in the glucagon signaling pathway, insulin resistance, the AMPK signaling pathway, and fructose and mannose metabolism (Figure 5F and Supplementary Table 3).

**FIGURE 6:**
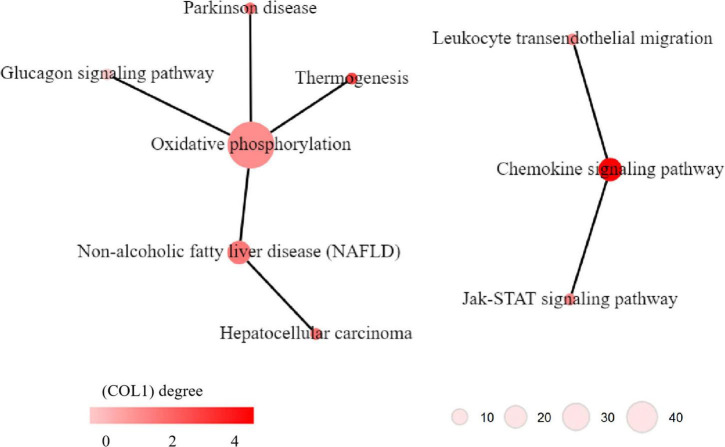
Kyoto Encyclopedia of Genes and Genomes (KEGG) pathway networks of dysregulated pathways in major depressive disorder (MDD). The circles indicate KEGG pathways. The edge between two pathways indicates that there is a relationship between the two pathways. The color of the circles indicates the degree of the pathways, i.e., how many edges linking to this pathways.

KEGG pathway network analysis was performed to explore the relationship between the significant pathways identified in KEGG pathway enrichment analysis. The results indicated that two networks were identified. One network contains six pathways, i.e., glucagon signaling pathway, thermogenesis, Parkinson’s disease, oxidative phosphorylation, non-alcoholic fatty liver disease (NAFLD) and hepatocellular carcinoma; the oxidative phosphorylation pathway had the largest degree in this pathway network (Figure 6 left), which was critical for energy metabolism. Another network contains three pathways, i.e., Jak-STAT signaling pathway, chemokine signaling pathway, and leukocyte transendothelial migration, the chemokine signaling pathway had the largest degree in this pathway network (Figure 6 right), which suggested that inflammation might involve in MDD.

## Discussion

In this study, a whole transcriptome sequencing was performed on the peripheral blood of MDD and matched HCs. The differentially expressed mRNAs, lncRNAs, miRNAs, and circRNAs, the dysregulated co-expression modules of RNA transcripts and the ceRNA network in MDD compared to HCs were identified. We also explored biological processes associated with dysregulation of transcriptome profiles by employing KEGG pathway analysis.

The ceRNA network indicated that 7 ceRNAs, including 4 lncRNAs and 3 circRNAs, might bind to 14 miRNAs and regulate the expression of 33 mRNAs. The identified lncRNA/circRNA-associated ceRNA network would offer novel insights into the pathophysiology of MDD and thus suggest novel treatments for the disease. In this network, hsa-let-7a-5p, hsa-let-7b-5p, hsa-let-7d-5p, hsa-let-7g-5p, and hsa-let-7i-5p belong to the let-7 family, which is the most highly expressed miRNA in the human brain and exerts a pivotal action on neuronal differentiation and maturation during neurodevelopment and on neurogenesis and neuronal plasticity functions in the adult brain (35, 36). Previous studies showed that hsa-let-7a-5p, hsa-let-7d-5p, and hsa-let-7g-5p were significantly dysregulated in MDD (37, 38) and gradually recovered to normal after 12 weeks of treatment with escitalopram, which suggested that the three miRNAs may be involved in both the pathogenesis of MDD and the effects of antidepressant drugs (37, 39). This is consistent with accumulated evidence that depression is associated with synaptic deficits, while neuroinflammation may influence neuronal activity as well as synaptic plasticity and contribute to neurobiological changes underlying MDD (40, 41). Second, let-7 is a family of miRNAs known to be involved in metabolic control (42). Previous studies showed that hsa-let-7a-5p was dysregulated in individuals with prediabetes and T2D (43), and the levels increased after 12 months of diabetic medication (44). The expression of hsa-let-7b-5p, hsa-let-7i-5p, and hsa-miR-92a-3p was significantly decreased in obese patients after bariatric surgery (45). Plasma hsa-let-7d-5p, hsa-let-7g-5p, and hsa-let-7i-5p were significantly dysregulated in Alzheimer’s disease (AD), and their target genes involved in the disturbance of multiple enzymatic pathways, including lipid metabolism, could play a role in AD etiology (46–48). Let-7b is also involved in inflammation and the immune response by regulating the activation of NF-kB and mediating the down-regulation of IL-6 in macrophages (49, 50). Researchers reported that hsa-let-7b-5p was significantly dysregulated in patients with active Crohn’s disease compared with healthy controls and was correlated with serum C-reactive protein (CRP) levels (51, 52). Finally, other miRNAs were also associated with metabolism, inflammation and immunity, and oxidative phosphorylation. For instance, hsa-miR-25-3p and hsa-miR-92a-3p are related to type 1 diabetes and systemic lupus erythematosus (SLE), respectively (53, 54). While hsa-miR-199a-3p was shown to be able to effectively distinguish schizophrenia or Parkinson’s disease (PD) from healthy controls, its target genes were mostly enriched in the FoxO signaling pathway, which is a potential mediator in the pathogenesis of depression (55–57). These pieces of evidence suggested that RNAs in the network were involved in sugar and lipid metabolism, inflammation and immunity, and oxidative phosphorylation, which might play an important role in the occurrence and development of depression.

Interpretation of biological readouts of transcriptome profiles is one of the challenges in transcriptome analyses. In this study, KEGG pathway network analysis was applied for intergradation of the results of KEGG pathway enrichment analysis, which was helpful for exploration of the potential biological mechanism underlying MDD. Two pathway networks were identified, and the oxidative phosphorylation pathway and chemokine signaling pathway have the most connection to other pathways in the two networks, which may suggest the critical roles of the two pathways in the pathophysiology of MDD. The oxidative phosphorylation pathway is widely involved in energy metabolism processes. It is known that the brain requires a continuous supply of energy in the form of ATP, most of which is predominantly generated within mitochondria by oxidative phosphorylation of glucose *via* the tricarboxylic acid (TCA) cycle (58). Previous studies also indicated that alterations in the oxidative phosphorylation system might represent the pathophysiological basis of MDD, and mitochondrial energy metabolism could be considered one of the major mechanisms underlying chronic corticosterone (CORT)-mediated depression (59, 60). And abnormal energy metabolism is considered to be one of the important mechanisms for the occurrence and development of depression (61). In addition, some researchers suggested that MDD and AD might share a common pathological basis, and MDD was considered to be a precursor of AD (62). Evidence has been accumulating that impaired brain energetics are involved in the cause and progression of neurodegenerative disorders of aging, especially AD (63, 64). The chemokine signaling pathway is a critical pathway that participates in inflammation. Accumulating evidence has confirmed the association between chronic inflammation and MDD, which is manifested by increased levels of multiple proinflammatory cytokines, such as interleukin (IL)-2, IL-6, CCL4, and CCL8 (65). Despite the considerable heterogeneity of experimental samples and methodologies, previous studies have demonstrated the important roles of chemokines in the pathophysiology of MDD (66). Chemokines could be considered potential peripheral markers and treatment targets for MDD. Therefore, the roles of oxidative phosphorylation-related energy metabolism and chemokine signaling pathway-related inflammation in MDD deserve refining study in the future.

Our research has some limitations. First, the sample size was relatively small due to the high costs of sequencing, which may result in insufficient sample representativeness. Second, we could not exclude the long-term effect of antidepressants on the results even though patients had not taken any antidepressant medication for at least 3 months before enrollment. Therefore, large sample studies are warranted in the future.

## Conclusion

In conclusion, our results suggested that RNAs in peripheral blood mainly affected metabolism, inflammation and immunity, and oxidative phosphorylation, thereby being involved in the pathophysiology of MDD. The results will be helpful for better understanding the pathophysiology of MDD and provide novel clues for exploring molecular biomarkers.

## Data Availability Statement

The datasets presented in this study can be found in online repositories. The names of the repository/repositories and accession number(s) can be found in the article/Supplementary Material.

## Ethics Statement

The studies involving human participants were reviewed and approved by the Ethics Committee of West China Hospital of Sichuan University. The patients/participants provided their written informed consent to participate in this study.

## Author Contributions

YW, JW, TC, TL, and XM contributed to the conception and design of the review. YW, XY, MW, YKD, and YD contributed to the data collection. YW, JW, LZ, and RN performed the statistical analysis. YW and JW wrote the first draft of the manuscript. XM critically revised the manuscript. All authors contributed to the article and approved the submitted version.

## Conflict of Interest

The authors declare that the research was conducted in the absence of any commercial or financial relationships that could be construed as a potential conflict of interest.

## Publisher’s Note

All claims expressed in this article are solely those of the authors and do not necessarily represent those of their affiliated organizations, or those of the publisher, the editors and the reviewers. Any product that may be evaluated in this article, or claim that may be made by its manufacturer, is not guaranteed or endorsed by the publisher.

## Footnotes

1
https://www.cs.cornell.edu/people/tj/svm_light/old/svm_light_v6.01.html

2
https://www.omicshare.com/tools
