# Supplementary material for: A Whole Transcriptome Analysis in Peripheral Blood Suggests That Energy Metabolism and Inflammation Are Involved in Major Depressive Disorder
Source: Front Psychiatry. 2022 May 13;13:907034. doi: 10.3389/fpsyt.2022.907034 (PMC9136012; doi:10.3389/fpsyt.2022.907034)
Supplement: Supplementary file 1 [file Data_Sheet_1.docx]

Table S1. TOP 20 pathways in KEGG pathway enrichment analysis of differential expression RNA transcripts

| **Names of Pathways** | **Hits** | **Number of genes in Pathways** | **Percent (%)** | **P value** | **FDR** | **Pathway ID** |
| --- | --- | --- | --- | --- | --- | --- |
| Parkinson disease | 22 | 1149 | 5.51 | 1.01×10^-6^ | 3.72×10^-4^ | ko05012 |
| Ribosome | 22 | 1222 | 5.51 | 4.12×10^-6^ | 4.98×10^-4^ | ko03010 |
| Thermogenesis | 30 | 2465 | 7.51 | 1.28×10^-4^ | 0.011 | ko04714 |
| Oxidative phosphorylation | 17 | 1071 | 4.26 | 2.08×10^-4^ | 0.013 | ko00190 |
| Renal cell carcinoma | 13 | 694 | 3.25 | 2.45×10^-4^ | 0.013 | ko05211 |
| Hepatocellular carcinoma | 22 | 1764 | 5.51 | 7.32×10^-4^ | 0.033 | ko05225 |
| Non-alcoholic fatty liver disease (NAFLD) | 20 | 1586 | 5.01 | 0.001 | 0.042 | ko04932 |
| Chemokine signaling pathway | 20 | 1615 | 5.01 | 0.001 | 0.046 | ko04062 |
| Jak-STAT signaling pathway | 17 | 1287 | 4.26 | 0.001 | 0.047 | ko04630 |
| Proteoglycans in cancer | 24 | 2180 | 6.01 | 0.002 | 0.061 | ko05205 |
| Platinum drug resistance | 12 | 814 | 3.01 | 0.003 | 0.074 | ko01524 |
| FoxO signaling pathway | 18 | 1507 | 4.51 | 0.003 | 0.074 | ko04068 |
| Alzheimer disease | 20 | 1760 | 5.01 | 0.003 | 0.074 | ko05010 |
| Chronic myeloid leukemia | 12 | 835 | 3.01 | 0.003 | 0.074 | ko05220 |
| Apoptosis | 19 | 1679 | 4.76 | 0.004 | 0.085 | ko04210 |
| B cell receptor signaling pathway | 11 | 764 | 2.75 | 0.005 | 0.092 | ko04662 |
| Bacterial invasion of epithelial cells | 13 | 1024 | 3.25 | 0.007 | 0.117 | ko05100 |
| T cell receptor signaling pathway | 13 | 1038 | 3.25 | 0.008 | 0.119 | ko04660 |
| Alcoholism | 15 | 1293 | 3.75 | 0.009 | 0.119 | ko05034 |

Table S2 TOP 20 pathways of modules

| **Modules** | **Names of Pathways** | **Hits** | **Number of genes in Pathways** | **Percent (%)** | **P value** | **FDR** | **Pathway ID** |
| --- | --- | --- | --- | --- | --- | --- | --- |
| Blue | Chemokine signaling pathway | 23 | 1615 | 6.02 | 4.46×10^-5^ | 0.012 | ko04062 |
|  | T cell receptor signaling pathway | 17 | 1038 | 4.45 | 8.59×10^-5^ | 0.012 | ko04660 |
|  | Leukocyte transendothelial migration | 17 | 1209 | 4.45 | 5.00×10^-4^ | 0.042 | ko04670 |
|  | Sulfur metabolism | 4 | 69 | 1.05 | 6.03×10^-4^ | 0.042 | ko00920 |
|  | Pathways in cancer | 46 | 5154 | 12.04 | 0.001 | 0.056 | ko05200 |
|  | Legionellosis | 10 | 558 | 2.62 | 0.001 | 0.056 | ko05134 |
|  | Shigellosis | 13 | 884 | 3.40 | 0.001 | 0.056 | ko05131 |
|  | Measles | 17 | 1347 | 4.45 | 0.001 | 0.056 | ko05162 |
|  | Regulation of actin cytoskeleton | 22 | 2058 | 5.76 | 0.002 | 0.089 | ko04810 |
|  | Salmonella infection | 13 | 1021 | 3.40 | 0.005 | 0.142 | ko05132 |
|  | Fc gamma R-mediated phagocytosis | 12 | 969 | 3.14 | 0.008 | 0.217 | ko04666 |
|  | Hepatocellular carcinoma | 18 | 1764 | 4.71 | 0.010 | 0.235 | ko05225 |
|  | Cytokine-cytokine receptor interaction | 16 | 1536 | 4.19 | 0.012 | 0.235 | ko04060 |
|  | Bacterial invasion of epithelial cells | 12 | 1024 | 3.14 | 0.012 | 0.235 | ko05100 |
|  | Pathogenic Escherichia coli infection | 8 | 556 | 2.09 | 0.013 | 0.235 | ko05130 |
|  | Apoptosis | 17 | 1679 | 4.45 | 0.013 | 0.235 | ko04210 |
|  | Osteoclast differentiation | 17 | 1755 | 4.45 | 0.019 | 0.301 | ko04380 |
|  | Acute myeloid leukemia | 9 | 721 | 2.36 | 0.020 | 0.301 | ko05221 |
|  | Proteoglycans in cancer | 20 | 2180 | 5.24 | 0.020 | 0.301 | ko05205 |
|  | Breast cancer | 13 | 1248 | 3.40 | 0.023 | 0.326 | ko05224 |
| Purple | Fatty acid degradation | 3 | 524 | 4.92 | 0.011 | 0.652 | ko00071 |
|  | Cytosolic DNA-sensing pathway | 3 | 591 | 4.92 | 0.015 | 0.652 | ko04623 |
|  | Influenza A | 6 | 2360 | 9.84 | 0.017 | 0.652 | ko05164 |
|  | Fatty acid metabolism | 3 | 698 | 4.92 | 0.024 | 0.652 | ko01212 |
|  | Aminoacyl-tRNA biosynthesis | 3 | 707 | 4.92 | 0.024 | 0.652 | ko00970 |
|  | Measles | 4 | 1347 | 6.56 | 0.031 | 0.686 | ko05162 |
|  | Protein processing in endoplasmic reticulum | 4 | 1852 | 6.56 | 0.081 | 0.896 | ko04141 |
|  | Amyotrophic lateral sclerosis (ALS) | 2 | 538 | 3.28 | 0.082 | 0.896 | ko05014 |
|  | N-Glycan biosynthesis | 2 | 556 | 3.28 | 0.087 | 0.896 | ko00510 |
|  | Glycosaminoglycan biosynthesis - chondroitin sulfate / dermatan sulfate | 1 | 105 | 1.64 | 0.088 | 0.896 | ko00532 |
|  | NOD-like receptor signaling pathway | 4 | 1923 | 6.56 | 0.090 | 0.896 | ko04621 |
|  | Nucleotide excision repair | 2 | 572 | 3.28 | 0.091 | 0.896 | ko03420 |
|  | TNF signaling pathway | 3 | 1212 | 4.92 | 0.092 | 0.896 | ko04668 |
|  | Nitrogen metabolism | 1 | 121 | 1.64 | 0.101 | 0.896 | ko00910 |
|  | purpEpstein-Barr virus infection | 5 | 2957 | 8.19 | 0.120 | 0.896 | ko05169 |
|  | Cardiac muscle contraction | 2 | 800 | 3.27 | 0.157 | 0.896 | ko04260 |
|  | Protein export | 1 | 204 | 1.63 | 0.165 | 0.896 | ko03060 |
|  | RIG-I-like receptor signaling pathway | 2 | 842 | 3.27 | 0.170 | 0.896 | ko04622 |
|  | IL-17 signaling pathway | 2 | 866 | 3.27 | 0.178 | 0.896 | ko04657 |
|  | Salivary secretion | 2 | 928 | 3.27 | 0.198 | 0.896 | ko04970 |
| Turquoise | Hepatocellular carcinoma | 27 | 1764 | 5.27 | 3.95×10^-4^ | 0.116 | ko05225 |
|  | T cell receptor signaling pathway | 18 | 1038 | 3.51 | 9.18×10^-4^ | 0.116 | ko04660 |
|  | Acute myeloid leukemia | 14 | 721 | 2.73 | 0.001 | 0.116 | ko05221 |
|  | NF-kappa B signaling pathway | 17 | 1037 | 3.32 | 0.002 | 0.120 | ko04064 |
|  | Renal cell carcinoma | 13 | 694 | 2.539 | 0.002 | 0.120 | ko05211 |
|  | Parkinson disease | 18 | 1149 | 3.516 | 0.002 | 0.120 | ko05012 |
|  | NOD-like receptor signaling pathway | 26 | 1923 | 5.078 | 0.002 | 0.120 | ko04621 |
|  | Legionellosis | 11 | 558 | 2.148 | 0.003 | 0.126 | ko05134 |
|  | B cell receptor signaling pathway | 13 | 764 | 2.539 | 0.005 | 0.157 | ko04662 |
|  | Chemokine signaling pathway | 22 | 1615 | 4.297 | 0.005 | 0.157 | ko04062 |
|  | MAPK signaling pathway | 38 | 3375 | 7.422 | 0.007 | 0.209 | ko04010 |
|  | mRNA surveillance pathway | 16 | 1091 | 3.125 | 0.008 | 0.209 | ko03015 |
|  | TNF signaling pathway | 17 | 1212 | 3.32 | 0.010 | 0.233 | ko04668 |
|  | Thermogenesis | 29 | 2465 | 5.664 | 0.010 | 0.233 | ko04714 |
|  | Hepatitis B | 22 | 1738 | 4.297 | 0.011 | 0.233 | ko05161 |
|  | Pathways in cancer | 52 | 5154 | 10.156 | 0.015 | 0.269 | ko05200 |
|  | Shigellosis | 13 | 884 | 2.539 | 0.016 | 0.269 | ko05131 |
|  | Toll-like receptor signaling pathway | 15 | 1079 | 2.93 | 0.016 | 0.269 | ko04620 |
|  | Apelin signaling pathway | 18 | 1388 | 3.516 | 0.017 | 0.269 | ko04371 |
|  | VEGF signaling pathway | 10 | 623 | 1.953 | 0.019 | 0.287 | ko04370 |
| Yellow | Porphyrin and chlorophyll metabolism | 9 | 382 | 4.072 | 4.69×10^-6^ | 0.001 | ko00860 |
|  | Glycine, serine and threonine metabolism | 6 | 428 | 2.715 | 0.002 | 0.326 | ko00260 |
|  | Thiamine metabolism | 3 | 139 | 1.357 | 0.010 | 0.617 | ko00730 |
|  | Purine metabolism | 12 | 1769 | 5.43 | 0.012 | 0.617 | ko00230 |
|  | Endocytosis | 17 | 2948 | 7.692 | 0.014 | 0.617 | ko04144 |
|  | Alanine, aspartate and glutamate metabolism | 4 | 299 | 1.81 | 0.016 | 0.617 | ko00250 |
|  | Phosphonate and phosphinate metabolism | 2 | 64 | 0.905 | 0.018 | 0.617 | ko00440 |
|  | Protein processing in endoplasmic reticulum | 11 | 1852 | 4.977 | 0.037 | 0.891 | ko04141 |
|  | mTOR signaling pathway | 10 | 1637 | 4.525 | 0.039 | 0.891 | ko04150 |
|  | Longevity regulating pathway - mammal | 8 | 1211 | 3.62 | 0.042 | 0.891 | ko04211 |
|  | Mitophagy - animal | 7 | 1001 | 3.167 | 0.043 | 0.891 | ko04137 |
|  | AMPK signaling pathway | 9 | 1453 | 4.072 | 0.045 | 0.891 | ko04152 |
|  | Oocyte meiosis | 8 | 1244 | 3.62 | 0.048 | 0.891 | ko04114 |
|  | African trypanosomiasis | 3 | 284 | 1.357 | 0.063 | 0.999 | ko05143 |
|  | Non-homologous end-joining | 2 | 132 | 0.905 | 0.067 | 0.999 | ko03450 |
|  | Phosphatidylinositol signaling system | 7 | 1142 | 3.167 | 0.076 | 0.999 | ko04070 |
|  | Autophagy - animal | 9 | 1658 | 4.072 | 0.087 | 0.999 | ko04140 |
|  | Ubiquitin mediated proteolysis | 8 | 1419 | 3.62 | 0.088 | 0.999 | ko04120 |
|  | Amyotrophic lateral sclerosis (ALS) | 4 | 538 | 1.81 | 0.095 | 0.999 | ko05014 |
|  | Adipocytokine signaling pathway | 6 | 996 | 2.715 | 0.102 | 0.999 | ko04920 |

Table S3 TOP 20 pathways of ceRNA network

| **Names of Pathways** | **Hits** | **Number of genes in Pathways** | **Percent (%)** | **P value** | **FDR** | **Pathway ID** |
| --- | --- | --- | --- | --- | --- | --- |
| Glucagon signaling pathway | 5 | 1176 | 20.83 | 4.61×10^-5^ | 0.002 | ko04922 |
| Insulin resistance | 5 | 1401 | 20.83 | 1.06×10^-4^ | 0.002 | ko04931 |
| AMPK signaling pathway | 5 | 1453 | 20.83 | 1.26×10^-4^ | 0.002 | ko04152 |
| Fructose and mannose metabolism | 3 | 400 | 12.50 | 3.58×10^-4^ | 0.005 | ko00051 |
| Adherens junction | 3 | 1115 | 12.50 | 0.006 | 0.071 | ko04520 |
| Phosphatidylinositol signaling system | 3 | 1142 | 12.50 | 0.007 | 0.071 | ko04070 |
| Malaria | 2 | 394 | 8.33 | 0.008 | 0.072 | ko05144 |
| Bladder cancer | 2 | 448 | 8.33 | 0.010 | 0.080 | ko05219 |
| Thyroid hormone signaling pathway | 3 | 1465 | 12.50 | 0.013 | 0.086 | ko04919 |
| Vasopressin-regulated water reabsorption | 2 | 544 | 8.33 | 0.015 | 0.086 | ko04962 |
| Cocaine addiction | 2 | 549 | 8.33 | 0.015 | 0.086 | ko05030 |
| TGF-beta signaling pathway | 2 | 603 | 8.33 | 0.018 | 0.094 | ko04350 |
| Insulin signaling pathway | 3 | 1747 | 12.50 | 0.022 | 0.103 | ko04910 |
| Amphetamine addiction | 2 | 751 | 8.33 | 0.027 | 0.110 | ko05031 |
| p53 signaling pathway | 2 | 771 | 8.33 | 0.029 | 0.110 | ko04115 |
| PI3K-Akt signaling pathway | 4 | 3471 | 16.66 | 0.030 | 0.110 | ko04151 |
| Cell adhesion molecules (CAMs) | 3 | 2005 | 12.50 | 0.031 | 0.110 | ko04514 |
| Thyroid hormone synthesis | 2 | 825 | 8.33 | 0.033 | 0.110 | ko04918 |
| ECM-receptor interaction | 2 | 842 | 8.33 | 0.034 | 0.110 | ko04512 |
| Cortisol synthesis and secretion | 2 | 867 | 8.33 | 0.036 | 0.110 | ko04927 |
